# Narbenhernien: Epidemiologie, Evidenz und Leitlinien

**DOI:** 10.1007/s00104-023-01999-3

**Published:** 2023-12-11

**Authors:** R.H. Fortelny, U. Dietz

**Affiliations:** 1 Lehrstuhl für Allgemeinchirurgie, Medizinische Fakultät, Sigmund Freud PrivatUniversität Wien, Freudplatz 3, 1020 Wien, Österreich; 2 Chirurgie, Kantonsspital Olten, Olten, Schweiz

**Keywords:** Inzisionale Hernie, Risikofaktoren, Bauchdeckenverschluss, Kurzstichtechnik, Prophylaktisches Netz, Incisional hernia, Risk factors, Abdominal wall closure, Short stitch technique, Prophylactic mesh

## Abstract

**Hintergrund:**

Aus epidemiologischer Sicht erfolgt bei einem Drittel der Bevölkerung in den Industrieländern im Laufe des Lebens eine abdominelle Operation. Je nach Grad des patientInnen- wie auch eingriffsbezogenen Risikos ist das Auftreten von Narbenhernien in einem Bereich von bis zu 30 % im 2‑Jahres-Follow-up und sogar bis zu 60 % nach 5 Jahren verbunden. Neben den beeinflussenden Komorbiditäten ist die Art des chirurgischen Zuganges und die Verschlusstechnik von entscheidender Bedeutung.

**Ziel:**

Die deskriptive Darstellung einer evidenzbasierten Empfehlung zum Verschluss der Bauchdecke sowie einer prophylaktischen Netzaugmentation.

**Material und Methoden:**

Unter Einbeziehung der aktuellen Literatur und der bestehenden Leitlinien wurde eine übersichtliche Zusammenfassung erstellt.

**Ergebnisse:**

Das bekannte Risiko für das Auftreten von Narbenhernien gilt bei Vorliegen von Adipositas und Erkrankungen der Bauchaorta nach neuesten Studien auch für PatientInnen mit einem kolorektalen Eingriff und Vorliegen einer Rektusdiastase. Auf Basis hochrangig publizierter Daten ist die Kurzstichtechnik bei Laparotomien der Mittellinie im elektiven Setting mit hoher Evidenz als Standardverfahren zu bezeichnen. PatientInnen mit erhöhtem Risikoprofil sollten neben der Kurzstichtechnik eine prophylaktische Netzverstärkung, sei es in Onlay- oder Sublay-Technik, erhalten. Bei Notfalllaparotomien muss das individuelle Infektionsrisiko bezüglich der angewendeten Verschlusstechnik einbezogen werden.

**Schlussfolgerung:**

Die Vermeidung von Narbenhernien ist in erster Linie durch den minimal-invasiven Zugang der Laparoskopie zu erzielen. Zum Verschluss des am häufigsten angewendeten Mittellinienzuganges ist die Kurzstichtechnik und bei bestehenden Risikofaktoren zusätzlich eine Netzaugmentation zu empfehlen.

## Abstract (en)

**Background:**

From an epidemiological point of view, one third of the population in industrialized countries will undergo abdominal surgery during their lifetime. Depending on the degree of patient-related and procedure-related risks, the occurrence of incisional hernias is associated in a range of up to 30% at 2‑year follow-up and even up to 60% at 5 years. In addition to influencing comorbidities, the type of surgical approach and closure technique are of critical importance.

**Objective:**

To present a descriptive evidence-based recommendation for abdominal wall closure and prophylactic mesh augmentation.

**Material and methods:**

A concise summary was prepared incorporating the current literature and existing guidelines.

**Results:**

According to recent studies the recognized risk for the occurrence of incisional hernias in the presence of obesity and abdominal aortic diseases also applies to patients undergoing colorectal surgery and the presence of diastasis recti abdominis. Based on high-level published data, the short stitch technique for midline laparotomy in the elective setting has a high level of evidence to be a standard procedure. Patients with an increased risk profile should receive prophylactic mesh reinforcement, either onlay or sublay, in addition to the short stitch technique. In emergency laparotomy, the individual risk of infection with respect to the closure technique used must be included.

**Conclusion:**

The avoidance of incisional hernias is primarily achieved by the minimally invasive access for laparoscopy. For closure of the most commonly used midline approach, the short stitch technique and, in the case of existing risk factors, additionally mesh augmentation are recommended.

## Epidemiologie

Ein Drittel aller Menschen in den Industrieländern muss sich im Laufe des Lebens einer abdominellen Operation unterziehen [1]. Bezieht man sich auf die Gruppe der über 60-Jährigen steigt die Rate sogar über 43 %. Ventrale Narbenhernien verursachen erhebliche Morbiditäten für die betroffenen PatientInnen. Neben der Größenzunahme, die häufig eine Verschlechterung der Funktionsfähigkeit sowie der körperlichen Komponenten der gesundheitsbezogenen Lebensqualität und des Körperimages mit sich bringt [2], kann es auch zu schwer versorgbaren Hautdefekten kommen. Eine der schwerwiegendsten Komplikationen stellt aber die Einklemmung und Strangulation des Bruchsackinhaltes dar, die eine sofortige Notoperation erfordern. Bekanntermaßen sind die Kurz- und Langzeitergebnisse solcher Notfalleingriffe deutlich schlechter im Vergleich zur elektiven Narbenhernienchirurgie, diese selbst aber kann per se, in Abhängigkeit der Komplexität des Falles, ebenfalls mit Wundheilungsstörungen und Narbenhernienraten von jeweils von 20–30 % assoziiert sein [3].

Schätzungen nach werden in den Vereinigten Staaten jährlich 3,2 Mrd. Dollar für die chirurgische Versorgung ventraler Hernien ausgegeben. Im Falle von Komplikationen nach der chirurgischen Behandlung bewegen sich die Kosten einer ventralen Narbenhernie im Gesundheitssystem pro Fall in einer Höhe von 30.000 bis 210.000 Dollar [4].

Im Gegensatz zum allgemeinen Trend der minimal-invasiv laparoskopisch durchgeführten Operationen wird vor allem in der onkologischen viszeral-, gynäko- und urologischen Chirurgie nach wie vor die Mittellinienlaparotomie als häufigster Zugang in den Bauchraum gewählt. Ein weiterer über die Linea alba durchgeführter Eingriff betrifft die Chirurgie der Aorta abdominalis, vor allem im akuten Setting. Neben dem Platzbauch als gefürchtetste Frühkomplikation nach Mittellinienlaparotomie stehen vor allem die weitaus häufiger im Langzeitverlauf auftretenden Narbenhernien als Komplikation im Vordergrund.

## Inzidenz

In der Literatur wird die Inzidenz von Narbenhernien nach einer Mittelinienlaparotomie zwischen 11 und 20 % nach einer mittleren Nachbeobachtungszeit von 12 und 20 Monaten angegeben [5]. Betrachtet man dies unter dem Aspekt des endgültigen Prozentsatzes von Narbenhernien, so ist im zeitlichen Verlauf innerhalb der ersten 6 Monate mit etwa 31 % zu rechnen, mit ca. 55 % innerhalb eines Jahres, mit 75 % innerhalb von 2 Jahren und erst innerhalb von 5 Jahren sind 89 % laut einer retrospektiven Studie zu erwarten [6, 7]. Das bedeutet für wissenschaftliche Studien, zumindest einen 5‑Jahres-Nachbeobachtungszeitraum zu fordern, um die wahre Höhe der Inzidenz zu erfahren. Eine 3‑Jahres-Nachbeobachtung zweier Studien zeigte einen relativen Anstieg der Inzidenz von mehr als 60 % zwischen dem 1. und 3. Jahren (von 13,1 % auf 21,3 % [7]).

Langzeitdaten zeigen die bis dato deutlich unterschätzte Inzidenz ventraler Narbenhernien

Im Vergleich der Kurz- vs. Langstichtechnik in 3 randomisiert kontrollierten Studien (RCT; [8–10]) zum Bauchdeckenverschluss findet sich eine kumulative 1‑Jahres-Narbenhernienrate für das Kurzstichverfahren von 8,19 % vs. 16,83 % für das Langstichverfahren. Die kürzlich publizierten Follow-up-Daten des PRIMA-Trials [11] berichten einen Anstieg der 2‑Jahres-Narbenhernienrate von 30 % auf 53,4 % nach 5 Jahren für die Langstichtechnik ohne Netzverstärkung bei RisikopatientInnen (Body-Mass-Index [BMI] ≥ 27 kg/m^2^, abdominelles Aortenaneurysma [AAA]). Das entspricht einem beachtlichen Anstieg von 78 % im Vergleich zum Ausgangswert. Diese Langzeitdaten implizieren eindrücklich die bis dato deutlich unterschätzte Inzidenz ventraler Narbenhernien.

## Risikofaktoren

Höer et al. [7] untersuchten in einer retrospektiven Studie mit Einschluss von 2983 laparotomierten PatientInnen über einen Zeitraum von 10 Jahren 42 Einzelfaktoren für die Narbenhernienentstehung. Die multivariate Analyse ergab als relevante Einflussfaktoren: BMI ≥ 25 kg/m^2^, männliches Geschlecht, Rezidivinzision, maligne Erkrankung und Wundkontamination.

In einer prospektiven Studie von Goodenough et al. [11] wurden bei PatientInnen, die sich einer abdominellen Operation, offen oder laparoskopisch unterzogen, nach median 41 Monaten eine Narbenhernienrate von 13,9 % detektiert. Als 4 unabhängige Prädiktoren fanden sich: Laparotomie oder handassistierte Laparoskopie, chronisch obstruktive Lungenerkrankung und ein BMI von 25 kg/m^2^. Zu den Faktoren, die nicht prädiktiv waren, gehörten Alter, Geschlecht, ASA-Score (American Society of Anesthesioloigsts), Albumin, Immunsuppression, vorherige Operationen sowie Nahtmaterial oder -technik.

Wehrle et al. [12] berichteten in einer retrospektiven Studie über die Ergebnisse bei 2241 adipösen PatientInnen (BMI ≥ 30 kg/m^2^), die elektiv oder akut einer primären Mittellinienlaparotomie unterzogen wurden. Radiologisch konnte nach median 316 Tagen bei 51,9 % der PatientInnen eine Narbenhernie diagnostiziert werden. Die signifikant höchste Narbenhernienrate fand sich nach kolorektalen und allgemeinchirurgischen Eingriffen.

Mehrere Studien bestätigen die Evidenz der Adipositas als Risikofaktor

In den rezent publizierten 5‑Jahres-Follow-up-Daten des PRIMA-Trials [13] findet sich in der Risikogruppe der adipösen (BMI ≥ 27 kg/m^2^) mit Langstichtechnik versorgten PatientInnen ein Anstieg der Narbenhernienrate auf 48 % und bestätigt damit deutlich die Evidenz der Adipositas als Risikofaktor. Ebenfalls aus dieser dreiarmigen Studie zum elektiven Bauchdeckenverschluss ist das Risiko für die Gruppe der mit abdominellem Aortenaneurysma (AAA) operierten PatientInnen mit einer Narbenhernienrate von 62 % nach Langstichverschluss 5 Jahre postoperativ zu sehen.

Betrachtet man die sehr häufig gewählte Mittellinienlaparotomie als Zugang für die viszerale Akutchirurgie, so findet sich in einer kürzlich publizierten „matched case“ kontrollierten Studie von PatientInnen mit einer Platzbauchkomplikation in der multivariaten Analyse eine signifikante Assoziation mit dem simultanen Vorliegen einer Rektusdiastase [14]. In dieser Studie erfolgte der Laparotomieverschluss in Kurzstichtechnik mit einer Naht-Inzisions-Ratio von ≥ 4:1.

Die Prävalenz einer Rektusdiastase bei PatientInnen mit Leistenhernie wurde in einer multizentrischen Querschnittstudie mit Gegenüberstellung von proktologischen PatientInnen als Kontrollgruppe untersucht [15]. Die Analyse ergab einerseits, dass eine Rektusdiastase bei LeistenhernienpatientInnen in einer höheren Prävalenz im Vergleich zur Normpopulation vorlag, und andererseits, dass höheres Alter, erhöhter BMI und Diabetes mellitus als unabhängige Risikofaktoren für die Entwicklung einer Rektusdiastase zu werten waren.

Zusammenfassend können daher als Hochrisikogruppe für das Auftreten von Narbenhernien nach elektiver Mittellinienlaparotomie PatientInnen mit einem erhöhten BMI, PatientInnen mit einem AAA und PatientInnen, die einem kolorektalen Eingriff unterzogen werden, sowie PatientInnen mit gleichzeitigem Vorliegen einer Rektusdiastase angesprochen werden.

## Leitlinien

In den vergangenen Jahrzehnten gab es eine Debatte über die bestmögliche Verschlusstechnik und das zu bevorzugende Nahtmaterial. Nach der Metaanalyse von Diener et al. [16] im Jahr 2010 war offensichtlich, dass die fortlaufende Nahttechnik mit langfristig resorbierbarem Nahtmaterial beim elektiven Mittellinienverschluss zu bevorzugen ist. Analog zu Dieners Übersichtsarbeit fasste der 2017 veröffentlichte Cochran-Review [17] zusammen, dass monofiles, spät resorbierbares Nahtmaterial für den Bauchdeckenverschluss in Betracht gezogen werden sollte. Diese Reviews enthielten jedoch keine Schlussfolgerung oder Empfehlung bezüglich der Anwendung von Kurz- oder Langstichtechnik. Im Jahr 2017 folgte der MATCH-Review von Henriksen et al. [18], der die randomisierten kontrollierten Studien von Millbourn et al. [8] und die STITCH-Studie [9] in eine Subgruppenanalyse einbezog. Die kumulative Narbenhernienrate war in der Kurzstichtechnik mit 9,45 % signifikant niedriger als in der Langstichtechnik mit 19,30 % (*p* = 0,005). Die Schlussfolgerung aus dieser Metaanalyse war, dass die Verwendung eines langsam resorbierbaren Nahtmaterials und ein kontinuierlicher Nahtverschluss in Kurzstichtechnik zu einer signifikanten Verringerung der Narbenhernienrate im Vergleich zur Langstichtechnik führt.

Die einzige starke EHS/AHS-Empfehlung ist die Anwendung einer kontinuierlichen Nahttechnik

Das kürzlich veröffentlichte Update der Leitlinien der Europäischen und Amerikanischen Gesellschaft für Hernienchirurgie (EHS/AHS) für den Bauchdeckenverschluss [19] bezieht sich für den elektiven primären Laparotomieverschluss der Mittellinie im Wesentlichen auf zwei RCT von Millbourn und Deerenberg [8, 9]. Die Empfehlung zum primären, elektiven Mittellinienverschlusses enthält nur eine einzige starke Empfehlung, nämlich die Verwendung einer kontinuierlichen Nahttechnik. Weitere Kriterien, wie z. B. Kurz- oder Langstichtechnik und das Nahtmaterial betreffend, wurden „nur“ mit einem schwachen Empfehlungsgrad mangels ausreichender Evidenz im GRADE-Klassifikationssystem eingestuft [20].

Nach den rezent veröffentlichten 1‑Jahres-Daten der ESTOIH-Studie [10], die noch nicht in das Update der EHS einbezogen wurden, könnte sich jedoch die Evidenzlage in gewissem Maße verändern. Bezüglich des Platzbauchrisikos zeigte sich in den Kurzzeitergebnissen [21] im Cox-Proportional-Hazard-Modell ein signifikant geringeres Risiko für die Kurzstichtechnik (Hazard Ratio [HR] 0,1783 [0,0379–0,6617], *p* = 0,0115). Im 1‑Jahres-Follow-up fand sich für die Kurzstichtechnik eine Narbenhernienrate von 4,24 % und von 8,23 % für die Langstichtechnik (*p* = 0,14 %). Obwohl der Unterschied nicht signifikant war, waren die Ergebnisse für die Kurzstichtechnik im Trend und verglichen mit der Millbourn- und STITCH-Studie überzeugend der Langstichtechnik überlegen.

Wenngleich die Vermeidung bzw. Prävention möglicher Komplikationsquellen einzubeziehen sind, bleibt zumindest die chirurgische Verschlusstechnik als standardisiertes Verfahren der wesentlichste Faktor für eine unkomplizierte Wundheilung der Bauchdecke.

Trotz der allgemeinen Definition der Kurznahttechnik scheint diese in den drei genannten Studien doch leicht unterschiedlich interpretiert zu werden. Neben der Nahttechnik ist das verwendete Nahtmaterial in Kombination mit Nadelgröße, -form und -stärke ein weiterer wichtiger Faktor. Die Standardisierung ist daher ein unumgängliches Kriterium für eine Vergleichbarkeit wissenschaftlicher Studien [22]. Darüber hinaus bleibt der größte Risikofaktor für einen unkomplizierten Verlauf des Mittellinienverschlusses neben vielen anderen Faktoren immer noch der Chirurg und seine Expertise selbst, wie bereits aus einer Studie aus dem Jahr 1998 [23] und einer rezent publizierten Studie ersichtlich ist [24].

## Verhältnis Naht- zu Inzisionslänge und Nahtmaterial

Bisher wurden drei randomisierte, kontrollierte Studien über die Kurz- bzw. Langstichtechnik bei der Mittellinienlaparotomie mit einem 1‑Jahres-Follow-up veröffentlicht [8–10]. Obwohl sich die Protokolle in Hinsicht auf die angewendete Verschlusstechnik auf den ersten Blick nicht signifikant unterscheiden, sind dennoch die 1‑Jahres-Ergebnisse zwischen der Millbourn-Studie, der STITCH-Studie und der ESTOIH-Studie hinsichtlich der Infektions- und Narbenhernienrate deutlich unterschiedlich (Tab. 1 und 2). Eine spezifische Ursache dafür zu finden bzw. retrospektiv zu analysieren, scheint äußerst schwierig. Ein wichtiger Parameter für eine spezifische Analyse könnte das Verhältnis der Naht- zur Wundlänge in der Gruppe der Kurzstichtechniken sein. Auch wenn dieses Verhältnis kein absoluter Wert für die exakte Durchführung einer Kurzstichtechnik ist, da das Verhältnis von verwendetem Nahtmaterial zur Inzisionslänge nur ein indirektes Maß in Abhängigkeit von der Anzahl der Stiche, dem Stichabstand und dem Umfang darstellt, so ist es doch der wichtigste zu erfassende Parameter für die Verschlusstechnik. Im Vergleich der drei Studien lag der höchste Wert für die Kurzstichtechnik bei 5,7 in der Millbourn-Studie, gefolgt von 5,3 in der ESTOIH-Studie und 5,0 in der STITCH-Studie. Diese Unterschiede mögen auf den ersten Blick gering erscheinen, könnten aber mit den deutlich unterschiedlichen Raten an Narbenbrüchen zusammenhängen. Da Israelsson die Bedeutung dieses Naht-Inzisions-Verhältnisses in mehreren Studien eindeutig nachgewiesen hat [25–27], scheint die unterste Grenze für dieses Verhältnis bei der Kurzstichtechnik nicht bei, sondern über 4:1 bzw. sogar 5:1 zu liegen.

**Table 1:** 

Technik	Infektionsrate		
	Millbourn	STITCH	ESTOIH
Langstich	10,5 %	23 %	5,7 %
Kurzstich	5,2 % signifikant	20 % signifikant	3,7 % nichtsignifikant

**Table 2:** 

Technik	Narbenhernienrate		
	Millbourn	STITCH	ESTOIH
Langstich	18 %	21 %	8,23 %
Kurzstich	5,6 % signifikant	13 % signifikant	4,24 % nichtsignifikant

Ein weiterer beeinflussender Faktor könnten die Eigenschaften des Nahtmaterials sein. In der Millbourn-Studie und in der STITCH-Studie wurde ein identisches Nahtmaterial aus Polydiaxanon (PDS©, Johnson & Johnson, New Brunswick, New Jersey, United States) verwendet. In der STITCH-Studie wurde offenbar ein Polydiaxanon mit Triclosan-Beschichtung (PDS plus©, Johnson & Johnson, New Brunswick, New Jersey, United States) verwendet, um Infektionskomplikationen zu verringern. Die hohen Infektionsraten in der STITCH-Studie von mehr als 20 % in beiden Gruppen sind jedenfalls wesentlich höher im Vergleich zur Millbourn- und ESTOIH-Studie.

Elastizität und Resorptionszeit des Nahtmaterials beeinflussen den Heilungsprozess

In der ESTOIH-Studie wurde das Nahtmaterial Poly-4-hydroxybutyrat (Monomax©, B. Braun, Melsungen, Germany) verwendet. Die mit 90 % signifikant im Vergleich zu Polydiaxanon mit 50 % erhöhte Elastizität dieses Nahtmaterials [28] in Kombination mit einer extrem langen Resorptionszeit im Vergleich zu Polydiaxanon sind Eigenschaften, die für die langzeitige Unterstützung in der Heilungsphase des Mittellinienverschlusses mit Ausbildung einer stabilen Narbe von Vorteil zu sein scheinen.

Für die Kurzstichtechnik sollte eine Fadenstärke von 2/0 in Kombination mit einer kleinen, feinkalibrierten Nadel (z. B. HR 26) verwendet werden. Einer der wesentlichsten Aspekte einer ungestörten Wundheilung ist die Durchblutung des Gewebes, speziell wenn es sich um die Linea alba bzw. die Aponeurose des Musculus obliquus externus handelt. Die Verwendung einer kaliberstarken Nadel (z. B. HR 48), wie diese bei der Schlingennaht zum Einsatz kommt, in Kombination mit hohen Zugkräften (> 1 Kilopond) führt unweigerlich zu einer verminderten Durchblutung und zur potenziellen Gefahr eines Platzbauches oder einer Narbenhernie im Langzeitverlauf. Daher ist eines der wesentlichsten Kriterien der Kurzstichtechnik ein moderater, adaptiver Zug an der Naht, um Früh- oder Spätkomplikationen zu vermeiden. Zusätzlich muss beim Vorliegen einer Rektusdiastase der Stichabstand zur Mittellinie mit zumindest 8 oder 10 mm empfohlen werden, um der Gewebeschwäche der ausgedünnten Aponeurose entgegenzuwirken.

Die Synergie einer standardisierten Kurzstichtechnik mit einem hochelastischen und ultralanglebigen resorbierbaren Material kann hier zu einem potenziell positiven Effekt führen. Allerdings ist die adäquate Nahttechnik (s. Infobox), die mittels Hands-on-Workshop trainiert und standardisiert werden sollte, die wesentlichste Grundvoraussetzung für ein komplikationsfreies Ergebnis [22].

## Prophylaktische Netzverstärkung

Die etablierten Risikofaktoren für die Entwicklung von Narbenhernien, wie das Vorliegen einer kollagenen Stoffwechselstörung, BMI > 27 kg/m^2^, AAA und andere Komorbiditäten, wie z. B. kolorektale Eingriffe, sollten bei jeder Laparotomie berücksichtigt werden und beeinflussen das Verschlussverfahren. Der Einsatz prophylaktischer Netzverfahren wird beim Vorliegen dieser Risikofaktoren empfohlen.

In den Updates der EHS/AHS-Leitlinien zum Bauchdeckenverschluss [19] wird eine schwache Empfehlung für eine prophylaktische Netzverstärkung nach elektiver Mittellinienlaparotomie aufgrund niedriger Evidenz abgegeben. In gleicher Weise wird die Netzplatzierung in Onlay- oder retromuskulärer Position mit sehr niedrigem Evidenzniveau empfohlen. Für die Versorgung akuter Mittellinienlaparotomien sind in diesen Leitlinien aufgrund fehlender Evidenz keine Empfehlungen zu finden.

Die bedeutendste, multizentrisch randomisiert kontrollierte Studie (PRIMA-Trial [29]) für den elektiven Einsatz prophylaktischer Netze zeigt hoch signifikant die Vorteile einer Netzverstärkung in Onlay- oder retromuskulärer Position im Vergleich zur alleinigen in Langstichtechnik verschlossenen Laparotomie bei PatientInnen mit einem erhöhten BMI von ≥ 27 kg/m^2^ oder einem Eingriff zur Reparatur eines AAA. In den 2‑Jahres-Daten fanden sich die geringsten Narbenhernienraten nach Onlay-Netzverstärkung mit 13 %, gefolgt von retromuskulärer Netzposition mit 18 und 30 % für den Langstichverschluss. Die aktuell publizierten 5‑Jahres-Daten [13] bestätigen diese Ergebnisse mit 24,7 % für das Onlay-Verfahren, 29,8 % für das retromuskuläre Verfahren und 53,4 % für den primären Langstichverschluss. In der Subgruppenanalyse weist die Adipositasrisikogruppe eine Narbenhernienrate von 48 % auf, die AAA-Risikogruppe sogar von 62 %.

Bei Risikogruppen zeigen sich signifikante Vorteile einer prophylaktischen Netzverstärkung

Analog zu diesen Daten findet sich in der PRIMAAT-Studie [30] mit Einschluss von AAA-operierten PatientInnen nach 5 Jahren der Nachbeobachtung eine Narbenhernienrate von 49,2 % für die Langstichgruppe vs. 0 % für die mit einem retromuskulären Netz versorgten PatientInnen. Allerdings muss hier noch ein potenzielles Detektionsbias aufgrund fehlender Bildgebung berücksichtigt werden, da 39,4 % der Gruppe mit primärer Naht und 35,3 % der Netzgruppe nur klinisch nachuntersucht wurden.

Bei einer weiteren randomisiert kontrollierten Studie, die ausschließlich PatientInnen wegen eines elektiven kolorektalen Eingriffes einschloss [31], zeigte sich ein signifikanter Vorteil der prophylaktischen Netzverstärkung im Onlay-Verfahren mit 11,3 % vs. 31,5 % mit primärer Langstichtechnik in Bezug auf die Narbenhernieninzidenz im 2‑Jahres-Follow-up.

Stabilini et al. [32] untersuchten das Risiko für Narbenhernien im Rahmen offen und laparoskopisch durchgeführter kolorektaler Eingriffe in 91 einbezogenen Studien und fanden metaregressionsanalytisch folgende Prädiktoren: Mittellinienlaparotomien, Präparatextraktion per Mittellinie und Rückoperationsverschlussstellen.

In einem aktuell publizierten systematischen Review mit Einbezug von 12 RCT wurde eine signifikante Reduktion der Narbenhernieninzidenz bei prophylaktischer Netzverstärkung mit 11,1 % vs. 21,3 % (*p* < 0,001) detektiert, allerdings bestand ein hohes Risiko für ein Publikationsbias [33]. Das Infektionsrisiko an der Operationsstelle war ohne Signifikanz (9,1 % vs. 8,9 %; *p* = 0,118), jedoch erhöhte das prophylaktische Netz das Risiko für eine SSO (surgical site occurrence) (14,2 % vs. 8,9 %; *p* < 0,001).

Die Verwendung eines prophylaktischen Netzes im Rahmen einer Akutlaparotomie ergab in der Metaanalyse von Burns et al. [34] unter Einschluss zweier nichtrandomisierten Studien [35, 36] mit 4,38 % vs. 31,36 % (*p* = 0,0001) signifikant geringere Narbenhernienraten in den Netzgruppen. In beiden Studien wurde kein Zusammenhang zwischen Netz und Infektion oder enterokutaner Fistel festgestellt.

In der Zusammenfassung der vorliegenden Literatur lässt sich auch bei Berücksichtigung eines gewissen Publikationsbias ein eindeutiger Vorteil für die Anwendung prophylaktischer Netze, vorzugsweise im Onlay- oder Sublay-Verfahren im elektiven Operationssetting, ableiten. Die Netzprophylaxe bei Akutlaparotomien ist bei geringer Evidenz noch individuell in Zusammenhang mit den vorliegenden Risikofaktoren abzustimmen.

## Kommentare zu primärer Bauchdeckenverschlusstechnik und prophylaktischen Netzen

Auf Basis der aktuellen Evidenz stellt die fortlaufende Kurzstichtechnik mit ultralangsam resorbierbarem Nahtmaterial heute den Goldstandard für den Verschluss primärer elektiver Mittellinienlaparotomien dar. Beim Vorliegen patientInnenbezogener wie auch eingriffsassoziierter Risikofaktoren sollte eine prophylaktische Netzverstärkung in Betracht gezogen werden.

Die resultierende Evidenz zu prophylaktisch netzaugmentierenden Verfahren beruht in erster Linie auf randomisiert kontrollierten Studien, die den Mittellinienverschluss ausschließlich im Langstichverfahren konzipiert hatten [29–31]. Aus heutiger Sicht der signifikanten Vorteile der Kurzstichtechnik sollte jedoch in Zukunft, wie in bereits laufenden Studien umgesetzt, jede mediane Laparotomie mittels Kurzstichverfahren, unabhängig von Risikofaktoren, als Grundlage für einen optional netzgestützten Verschluss verwendet werden, um die Narbenhernienrate noch nachhaltiger zu senken.

## Kriterien für einen „tailored approach“

Das patientInnenbezogene Risikoprofil (BMI ≥ 27 kg/m^2^, Vorliegen eines AAA, Rektusdiastase) unter Berücksichtigung des durchzuführenden Eingriffes (z. B. kolorektaler Eingriff, abdomineller Aorteneingriff) wie auch der intraoperativen Situation (z. B. akut, elektiv, Kontaminationsgrad) ist die Grundlage für die Entscheidungsfindung, welcher Zugang und welche Art des Verschlusses gewählt werden sollte. Im Idealfall ist ein minimal-invasiver immer dem offenen Zugang vorzuziehen. Im Fall der Wahl für eine mediane Laparotomie, sei es elektiv wie auch bei Notfalleingriffen, gelten prinzipiell dieselben Kriterien, jedoch müssen im akuten Setting das patientInnenbezogene Risiko und der Kontaminationsgrad für die prophylaktische Netzimplantation individuell abgewogen werden.

### Infobox Kriterien der Kurzstichtechnik

- Nahtmaterial: monofil, elastisch, ultralangsam bzw. langsam resorbierbar
- Stärke: 2/0
- Nadel: HR 26
- Fortlaufend
- Ausschließlich Linea alba bzw. Aponeurose des M. obliquus externus
- Verhältnis Naht- zu Inzisionslänge ≥ 5:1
- Erster Stich > 1 cm außerhalb der Inzision (intaktes Gewebe)
- Stichabstand zur Inzision 5–8 mm
- Stich-zu-Stichabstand 5 mm
- Stichlänge ≤ 2,5 cm
- Adaptiver Zug an der Nahtreihe (≤ 1 kp)
- Cave → „button holes“
- Nahtstege sollen nach Fertigstellung sichtbar sein
- Optional selbstfixierender Knoten (Beginn: Röder-Knoten, Ende: Aberdeen-Knoten)

## Fazit für die Praxis

- Jede primäre, elektive Laparotomie der Mittellinie sollte in einer standardisierten Kurzstichtechnik mit einem Naht-Inzisions-Längenverhältnis von ≥ 5:1 erfolgen.
- Die Verwendung eines langzeitresorbierbaren, monofilen Nahtmaterials mit einer Stärke von 2/0 in Kombination mit einer kleinen Nadel (z. B. HR 24) wird dazu empfohlen.
- Die maximale Zugbelastung an der Naht sollte 1 Kp nicht überschreiten.
- Die prophylaktische Netzimplantation bei elektiven Mittellinienlaparotomien sollte bei PatientInnen mit hohem Risiko für die Entwicklung einer Narbenhernie in Betracht gezogen werden.
- Zur Risikogruppe zählen PatientInnen mit Adipositas, abdominellem Aortenaneurysma, bestehender Rektusdiastase und kolorektalem Eingriff.
- Die Implantation eines permanenten synthetischen Netzes kann in Onlay- oder retromuskulärer Position im Rahmen des Laparotomieverschlusses erfolgen.
- Die Kombination von Kurzstichtechnik und prophylaktischer Netzverstärkung sollte für den Verschluss der primären elektiven Mittellinienlaparotomie bei PatientInnen mit erhöhtem Risikoprofil pro futuro den Standard darstellen.
